# Regional activity alterations in Parkinson’s disease patients with anxiety disorders: A resting-state functional magnetic resonance imaging study

**DOI:** 10.3389/fnagi.2022.1055160

**Published:** 2022-12-16

**Authors:** Peiyao Zhang, Yanling Zhang, Yuan Luo, Lu Wang, Kang Wang

**Affiliations:** ^1^Department of Radiology, China-Japan Friendship Hospital, Beijing, China; ^2^Department of Neurology, China-Japan Friendship Hospital, Beijing, China

**Keywords:** Parkinson’s disease, anxiety, resting-state, functional MRI, regional homogeneity

## Abstract

**Background:**

Previous studies have revealed alteration of functional connectivity (FC) in Parkinson’s disease patients with anxiety (PD-A), but local brain activities associated with anxiety in Parkinson’s disease (PD) patients remain to be elucidated. Regional homogeneity (ReHo) analysis was employed to investigate alterations of regional brain activities in PD-A patients.

**Methods:**

Resting-state functional magnetic resonance imaging (rs-fMRI) data were acquired from 42 PD-A patients, 41 PD patients without anxiety (PD-NA), and 40 age-and gender-matched healthy control (HC) subjects. ReHo analysis was used to investigate the synchronization of neuronal activities in brain regions in the three groups. The relationship between ReHo value and anxiety score in the PD-A group was also investigated.

**Results:**

Parkinson’s disease patients with anxiety showed increased ReHo values in the bilateral frontal lobes, caudate nucleus, and anterior cingulate gyrus [Gaussian random field (GRF) correction, voxel size *p* < 0.01, cluster size *p* < 0.05], compared with PD-NA patients and HC subjects, but the ReHo values of the right cerebellar hemisphere and posterior cerebellar lobe decreased (GRF correction, voxel size *p* < 0.01, cluster size *p* < 0.05). The increased ReHo values of the right superior frontal gyrus (*r* = 0.633, *p* = 0.001) and anterior cingulate gyrus (*r* = 0.45, *p* = 0.01) were positively correlated with anxiety scores in PD-A patients.

**Conclusion:**

The development of PD-A may be associated with dysfunctional local activities in multiple brain regions, including the frontal cortex, cerebella, basal ganglia, and limbic system. Abnormal ReHo values in these brain regions may serve as neuroimaging markers for the early diagnosis of PD-A. The results suggest that using ReHo analysis to identify functional changes in core regions may advance our understanding of the pathophysiological mechanisms underlying PD-A.

## Introduction

Parkinson’s disease (PD) is one of the most prevalent chronic progressive neurodegenerative diseases in the elderly people (Rai et al., 2020, 2021; Rai and Singh, 2020). Anxiety is one of the most common non-motor symptoms of PD, with a prevalence of 25–49% in PD patients, much higher than that in the general population (Carey et al., 2021; Forbes et al., 2021). A considerable number of studies have indicated that anxiety disorders occur prior to motor symptoms, which might contribute to aggravating other motor symptoms, adversely affects the patient’s daily functioning, reduce the patient’s quality of life, increase the burden on the caregiver and expedite disease progression (Forbes et al., 2021; Wang et al., 2021). Even though PD with anxiety (PD-A) has a high prevalence and imposes a huge burden on patients’ daily lives, it is difficult to be recognized and diagnosed due to modest symptoms and a lack of standardized diagnostic criteria. Previous neurotransmitter studies have suggested that the dopaminergic uptake, norepinephrine transmission, and serotonergic neuron system dysfunction in the neuronal circuit such as the prefrontal cortex, striatum, cerebellum, and limbic system may be related to the occurrence of anxiety symptoms in PD patients (O’Callaghan et al., 2014; Moonen et al., 2017; Joling et al., 2018). However, the pathophysiological mechanism of PD-A is still confused. As a result, finding objective diagnostic markers to characterize the underlying neural mechanisms of PD-A has a lot of clinical significance.

In recent years, sophisticated neuroimaging techniques have been applied to investigate the neural mechanism of anxiety symptoms in Parkinson’s disease. Voxel-based morphometry and cortical thickness analyses visualized atrophy of the bilateral anterior cingulate cortex (ACC), left amygdala, bilateral prefrontal lobe, and bilateral cerebellum; neurotransmitter studies demonstrated that striatum dopaminergic uptake levels and noradrenergic transmission may be involved in the occurrence of PD-A (Saikiran, 2020; Carey et al., 2021). In particular, resting-state functional magnetic resonance imaging (rs-fMRI), which measures the functional connectivity (FC) between different brain regions at rest, has shown abnormalities in FC between the prefrontal cortex–limbic system, putamen–caudate nucleus, putamen–prefrontal cortex, and putamen–cerebellum in PD-A patients (Dan et al., 2017; Wang et al., 2017; Zhang et al., 2019; Carey et al., 2021). The bulk of published evidence concerning the clinical applications of rs-fMRI centers on its use in FC analysis of PD-A patients. However, there was a paucity of studies addressing whether anxious PD patients present abnormal local activities. Regional homogeneity (ReHo) is another rs-fMRI analysis, which was first proposed by Zang et al. (2004). It measures local functional synchronization of spontaneous neuronal activity between one voxel and its nearest neighbors and has been employed to assess local brain activities in various neuronal disorders (Fang et al., 2021; Du et al., 2022). In recent years, ReHo analysis has also been applied to detect the neural mechanism of non-motor symptoms of PD patients, including sleep disorders (Wen et al., 2016; Chen et al., 2019), hyposmia (Su et al., 2015) and cognitive impairment (Harrington et al., 2017; Xing et al., 2021). To date, only Wang et al. have proposed that PD-A patients have abnormal ReHo values in the left cerebellum and left medial frontal gyrus when compared to PD-NA patients and healthy controls +(HCs) (Wang et al., 2021). In this study, we enlarged the sample size of the subjects and selected PD patients with mild motor symptoms in stages 1–2.5, so as to explore the alterations of resting-state regional activities among PD-A patients, PD-NA patients, and HCs by using ReHo analysis. The purpose of the study is to provide accurate and objective imaging diagnostic indicators for detecting the pathophysiological mechanism of PD-A.

## Materials and methods

### Study design

This is a prospective study specifically designed to explore alteration in local activities of brain in Parkinson’s disease patients with anxiety. All participants underwent 1 MRI scan, and were instructed to relax and avoid movement during the scan.

### Participants

In this study, 42 PD-A patients, 41 PD-NA patients, and 40 healthy control (HC) subjects were recruited from the outpatient department of neurology at China-Japan Friendship Hospital between March 2019 and June 2021. The inclusion criteria for candidates of PD groups were: (1) be diagnosed with PD based on Movement Disorder Society (MDS) Clinical Diagnostic Criteria (Postuma et al., 2015) within 5 years, (2) modified Hoehn and Yahr (H-Y) stage of 1–2.5, and (3) Hamilton Anxiety Rating Scale (HAMA) score of ≥ 7 and < 7 for PD-A and PD-NA groups, respectively. The motor function of PD patients was assessed with the MDS modified version of the Unified Parkinson’s Disease Rating Scale motor examination (UPDRS-III). The exclusion criteria were (1) history of cerebrovascular diseases such as brain injuries, stroke, and hemorrhages, (2) presence of cognitive and mental disorders such as dementia, depression, alcohol, or drug dependence, (3) severe respiratory, digestive, and cardiovascular diseases, and (4) MRI contraindications such as metal implants and claustrophobia. All subjects were right-handed. This study was approved by the ethics committee of the China-Japan Friendship Hospital. All subjects provided written informed consent before participating in this study.

### Imaging data acquisition

MRI data were collected using a 3.0 T MR scanner (GE Discovery MR 750, United States) with an eight-channel head coil. All PD patients were suspended from anti-PD drugs for more than 48 h before scanning. All subjects were asked to keep their eyes closed and not to think about anything specific throughout the scan, but to avoid falling asleep. Three-dimensional T1-weighted images (3D-T1WI) were acquired with the following parameters: repetition time (TR) /echo time (TE) = 6.7 ms/2.5 ms, flip angle = 12°, slice thickness/gap = 1 mm/1 mm, slices = 192, field of view (FOV) = 256 mm × 256 mm. Resting-state fMRI images were acquired using the following parameters: TR/TE = 2,500 ms/30 ms, flip angle = 90°, slice thickness/gap = 3.5 mm/0, slices = 42, acquisition matrix = 64 × 64, FOV = 256 mm × 256 mm.

### Imaging data preprocessing and analysis

For image preprocessing, ReHo analysis were performed using the Data Processing & Analysis for Resting-State Brain Imaging (DPABI, V5.0)^1^ based on Statistical Parametrical Mapping 12 (SPM)^2^ in MATLAB 2014b (The Math Works, Inc., Natick, MA, United States). The main pre-processing steps were as follows: data format conversion, elimination of the first 10 time points, slice timing correction, head motion correction, spatial normalization, realignment nuisance covariate regression, removal of linear trends, and filtering in the frequency range of 0.01–0.1 Hz. The subjects with head motion and translation > 3 mm and rotation Angle > 3° were excluded.

Next, calculate Kendall’s coefficient concordance (KCC) of the time series of each brain voxel and its 26 neighboring voxels for each participant. The KCC of each voxel was then divided by the mean KCC of the whole brain to produce a standardized ReHo diagram. Finally, a Gaussian kernel of 6 mm full-width-half-maximum was used for spatial smoothing.

### Statistical analysis

The data of one PD-A patient and two PD-NA patient with excessive head motion were excluded, while the data of other participants were all meet the inclusion criteria. One-way analysis of variance (ANOVA) was applied to compare the age, MMSE and HAMA scores among the three groups. The Chi-square test was used to compare gender distributions. The illness duration, H-Y stage, and UPDRS scores between the two patient groups were compared by using Two-sample *t*-test. *p* < 0.05 was considered significantly different.

With age, gender, disease course, H&Y stage, and MDS-UPDRS-III as covariates, one-way ANOVA and post-hoc tests (Tukey–Kramer) were used to analyzed the differences of ReHo among the three groups and compare the differences of ReHo between each pair of the three groups. After that Pearson correlation analysis method was used to analyze the correlation between ReHo values in all brain regions of PD-A patients and HAMA scores with the same covariates. Comparisons above were corrected by Gaussian random field (GRF) correction, and threshold was set to *p* < 0.01 at the voxel level and *p* < 0.05 at the cluster level, two tailed. All the above analysis were performed by DPABI V5.0 based on SPM12 in MATLAB 2014b.

## Results

### Demographic and clinical characteristics

A total of 41 PD-A patients, 39 PD-NA patients, and 40 HC subjects were included in the final analysis. Table 1 presents all subjects’ demographic and clinical characteristics. There were no significant differences in terms of gender, age, and MMSE scores among the three groups. Similarly, the two PD groups did not differ in the disease duration, UPDRS-III score, or H-Y stage. While the HAMA score of the PD-A group was significantly higher than that of the other two groups (*p* < 0.05).

**Table 1:** Demographic and clinical characteristics.

Groups	PD-A (*n* = 41)	PD-NA (*n* = 39)	HC (*n* = 40)	*F/χ^2^/t*	*P*
Gender (M/F)	17/24	17/22	16/24	0.44	0.80^a^
Age (years)	65.54 ± 9.6	65.62 ± 10.23	60.97 ± 8.82	2.45	0.091^b^
Disease course (months)	39.31 ± 27.42	40.53 ± 35.37	-	-0.174	0.96^c^
MDS-UPDRS-III score	28.45 ± 12.92	21.80 ± 12.96	-	0.75	2.32^c^
Hoehn & Yahr stage	1.85 ± 0.59	1.73 ± 0.55	-	1.05	0.91^c^
HAMA score	11.26 ± 3.70	3.12 ± 2.0	2.50 ± 1.30	12.27	0.005^b*^
MMSE score	27.43 ± 1.58	28.22 ± 2.15	27.78 ± 1.66	1.99	0.14^b^

*χ*^2^ test; ^b^One-way ANOVA test; ^c^Two independent samples *t* test; ^∗^*p* < 0.05.

### Brain regional activities

**Figure 1:**
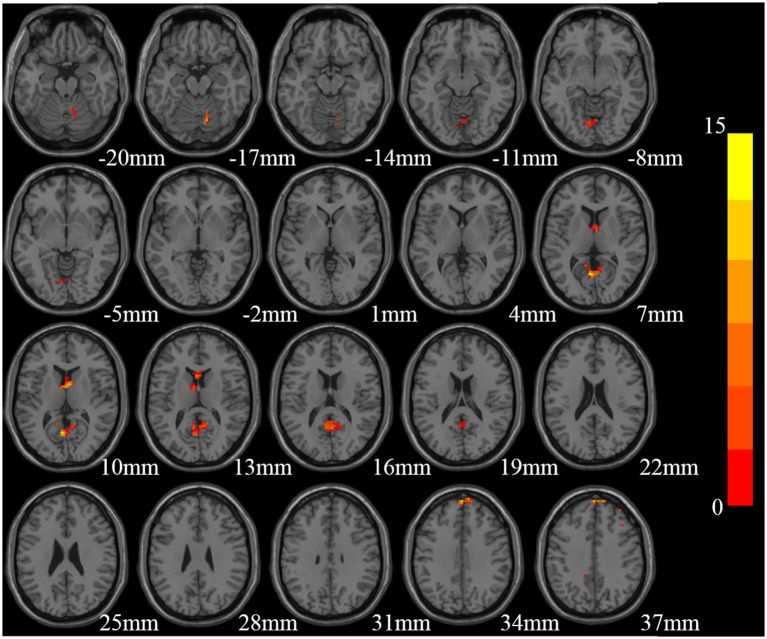
Brain regions with significant differences in Reho among the three groups [Gaussian random field (GRF) multiple comparison correction, *P* < 0.01 at the voxel level and *P* < 0.05 at the cluster level].

Significant ReHo differences were found mainly in the right cerebellum, cerebellar vermis, bilateral precuneus, bilateral caudate nucleus, bilateral superior frontal gyrus, and right middle frontal gyrus among the three groups (GRF correction, voxel size *p* < 0.01, cluster size *p* < 0.05, two tailed; Table 2; Figure 1).

**Table 2:** Brain regions with significant differences in ReHo among the three groups.

AAL	Coordinates of maximum			Cluster size	*F*
	X	Y	Z		
**Reho**					
Right Cbe6/CPL/Cbe4-5/Ver6	12	−69	−18	36	10.72
Bilateral PCUN	−3	−63	6	99	12.65
Bilateral CAU	6	3	9	44	11.60
Bilateral SFG	0	60	33	41	12.18
Bilateral PCUN	−9	−45	42	91	11.51
Right SFG/MFG	33	42	45	42	12.77

AAL, the automated anatomical labeling; Cbe, Cerebellum; Ver, Vermis; CPL, Cerebellum posterior lobe; PUCN, Precuneus; CAU, Caudate nucleus; SFG, Superior frontal gyrus; MFG, Middle frontal gyrus.

**Figure 2:**
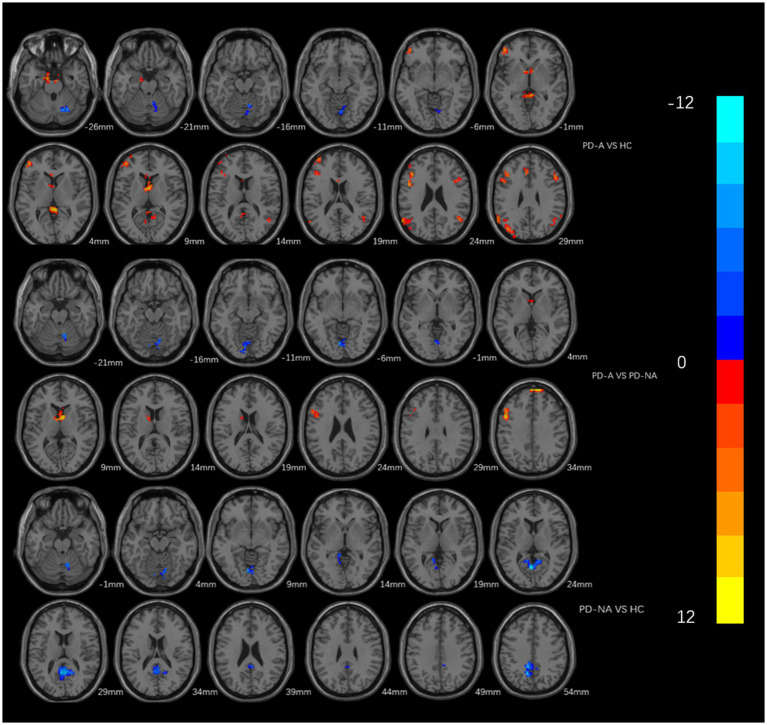
Brain areas which showing different Reho between every two groups. The red area represents the brain region with significant increase of ReHo (PD-A>HC, PD-A>PD-NA, PD-NA>HC), and the blue area vice versa (PD-A<HC, PD-A<PD-NA, PD-NA<HC) (GRF multiple comparison correction, *P* < 0.01 at the voxel level and *P* < 0.05 at the cluster level).

PD-A patients showed elevated ReHo in the bilateral frontal lobes, left parietal lobes, and bilateral precuneus compared with HC subjects, and reduced ReHo in the right cerebellum, cerebellar vermis, and cerebellum posterior lobe (GRF correction, voxel size *p* < 0.01, cluster size *p* < 0.05; Table 3; Figure 2). PD-NA patients had decreased ReHo in the right cerebellum, bilateral precuneus and limbic lobe, in contrast, no brain regions with increased ReHo compared with HC subjects (GRF correction, voxel size *p* < 0.01, cluster size *p* < 0.05, two tailed; Table 3; Figure 2).

**Table 3:** Brain areas with significant between-group ReHo differences.

AAL	Coordinates of maximum			Cluster size	*t*-value
	X	Y	Z		
**PD-A> HC**					
Left MFG /IFG	−42	30	21	251	3.91
Right MFG/SFG/IFG	18	24	54	249	4.03
Left ANG/IPL	−60	−60	27	234	4.02
Left MFG/ SFG	−6	30	33	168	4.23
Bilateral PCUN	0	−54	66	131	4.12
**PD-A< HC**					
Right Cbe6/Cbe4-5/CPL/Ver6	18	−66	−27	100	−3.54
**PD-A> PD-NA**					
Bilateral CAU /ACC	6	3	9	225	4.3
Bilateral SFG	12	60	36	104	4.11
Left MFG /IFG	−48	12	36	108	3.69
**PD-A< PD-NA**					
Right Cbe6/Cbe4-5/CPL/Ver6	12	−69	−18	117	−4.07
**PD-NA<HC**					
Bilateral PCUN/limbic lobe	−3	−63	6	477	−4.8

PD-A, PD with anxiety; PD-NA, PD without anxiety; HC, healthy control; AAL, the automated anatomical labeling; Cbe, Cerebellum; Ver, Vermis; CPL, Cerebellum posterior lobe; PUCN, Precuneus; CAU, Caudate nucleus; SFG, Superior frontal gyrus; MFG, Middle frontal gyrus; IFG, Inferior frontal gyrus; ANG, Angular gyrus; IPL, Inferior parietal gyrus; ACC, Anterior cingulate cortex.

In comparison between the two groups of PD, patients in the PD-A group showed higher ReHo in the bilateral frontal lobes, bilateral anterior cingulate gyrus, and bilateral caudate nucleus; and lower ReHo in the right cerebellum and cerebellum posterior lobe (GRF correction, voxel size *p* < 0.01, cluster size *p* < 0.05, two tailed; Table 3; Figure 2).

**Figure 3:**
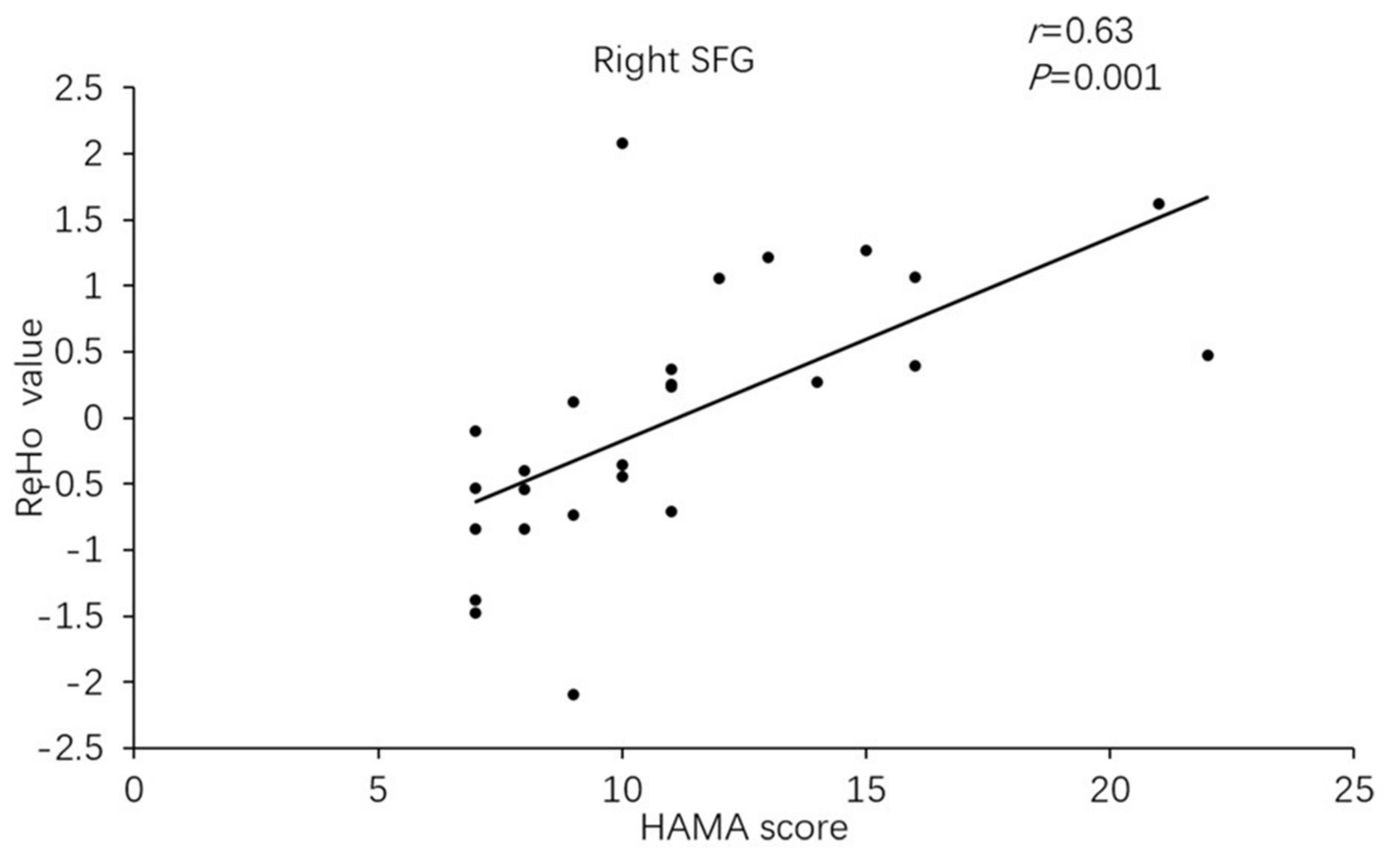
In Parkinson’s disease with anxiety (PD-A) patients, the ReHo value of the right superior frontal gyrus showed a positive correlation with Hamilton Anxiety Rating Scale (HAMA) scores.

**Figure 4:**
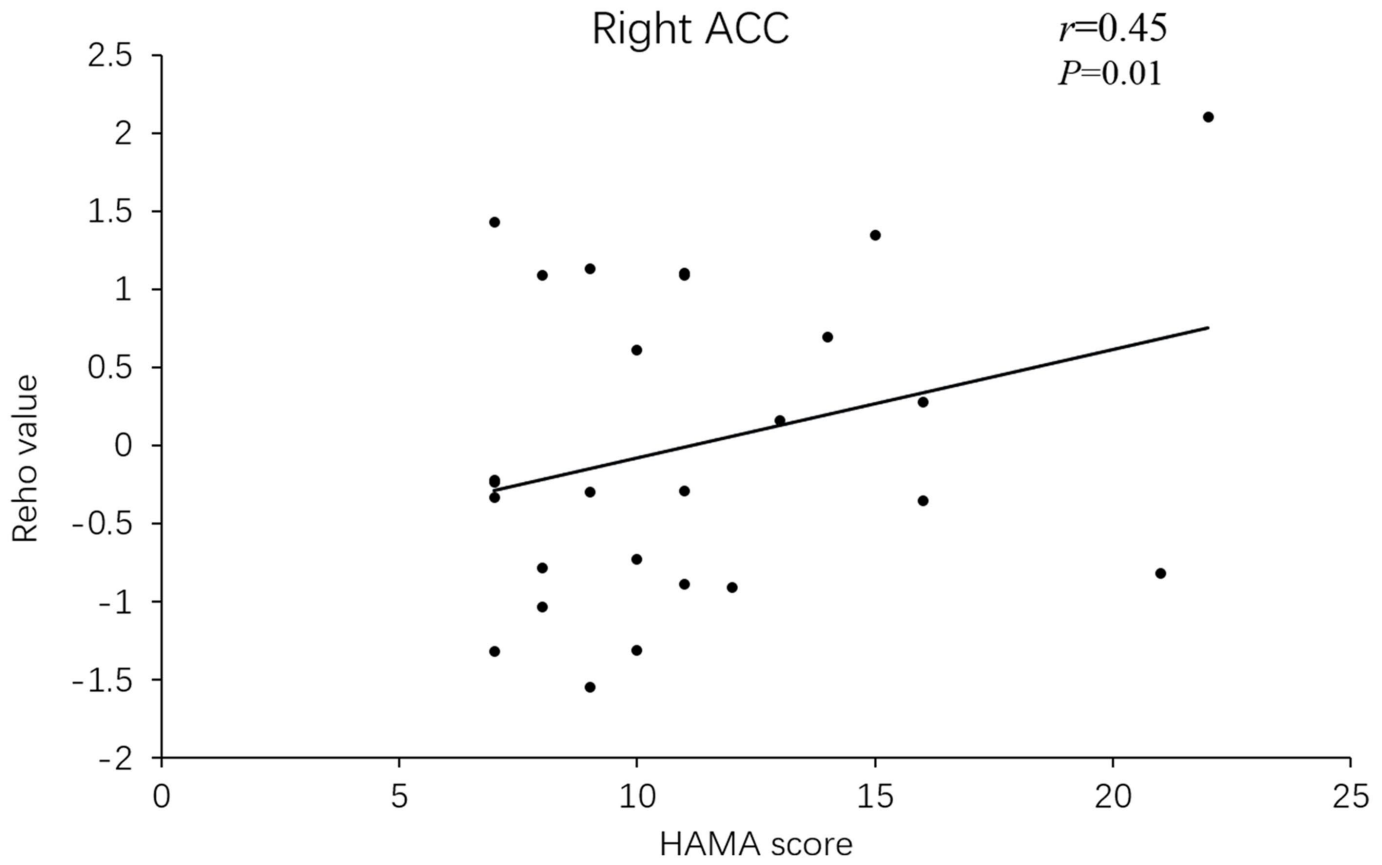
In PD-A patients, the ReHo value of the right anterior cingulate gyrus showed a positive correlation with HAMA scores.

Besides, the ReHo values in the right superior frontal gyrus (*r* = 0.633, *p* = 0.001) and ACC (*r* = 0.45, *p* = 0.01) of PD-A patients displayed positive correlations with HAMA scores in Pearson’s correlation analysis (Figures 3, 4).

## Discussion

In this study, we used ReHo analysis to investigate the association between anxiety symptoms and local brain activities in PD patients. Compared with HCs, PD-A patients showed altered ReHo in left frontal lobe, left parietal lobe, bilateral precuneus and right cerebellum. Compared with PD-NA patients, the altered ReHo was indicated in the bilateral frontal lobe, caudate nucleus, anterior cingulate gyrus of and right cerebellum. Furthermore, the elevated ReHo values of the right superior frontal gyrus and right anterior cingulate gyrus in PD-A patients were positively correlated with anxiety level.

The prefrontal lobe is the core brain area related to emotional disorders. Its functions include decision-making, emotional regulation, and social cognition (Hiser and Koenigs, 2018; Zhang et al., 2021). The inferior frontal gyrus is in the posterior part of the frontal lobe, which plays a vital role in the inhibition of brain response (Suda et al., 2020; Chaudhary et al., 2021). Dan et al. proposed that the severity of anxiety symptoms in PD patients was positively correlated with functional connectivity between the prefrontal cortex and middle and lower temporal lobes (Dan et al., 2017). Previous study have found that PD-A patients displayed increased low-frequency amplitude in the right orbitofrontal gyrus compared with normal controls and PD-NA patients (Wang et al., 2018). And the increased functional connectivity between the right orbitofrontal gyrus and parietal lobe was positively correlated with anxiety score (Carey et al., 2021). The results of our study were consistent with previous findings, indicating that increased ReHo in the frontal cortex of PD patients may lead to the spontaneous impairment in cognition and negative emotion processing, thus leading to anxiety disorders such as tension and fear.

Anterior cingulate cortex (ACC) is an important node of the limbic system which has extensive connections with multiple cortical and subcortical regions such as the prefrontal cortex, thalamus, and striatum. ACC is mainly related to self-concentration of attention, self-regulation of emotion, and situational memory extraction (Alexander et al., 2021; Scotti-Muzzi et al., 2021). Previous study has reported that ACC was involved in anxiety disorder caused by physiological diseases (Chaibi et al., 2021; Ren et al., 2021). Our study consistently found that the increased ReHo values in PD-A patients were positively correlated with the HAMA scores, suggesting that alteration of ReHo in ACC might cause the dysfunction of self-emotion and consciousness regulation in PD patients, and thus inducing anxiety symptoms. It also suggested that the anxiety symptoms in PD patients were not caused by the dysfunction of a single brain region but rather by the coordination dysfunction of multiple brain regions.

This study demonstrated that ReHo values in the bilateral caudate nuclei were higher in PD-A patients compared with PD-NA patients. The result is consistent with a new pathophysiological hypothesis proposed in recent years, the striatum is not only involved in the alteration of motor activity, but also in the regulation and processing of various emotions (Hiser and Koenigs, 2018). Previous studies have indicated that increased functional connectivity between the striatum and prefrontal cortex was positively correlated with HAMA score (Carey et al., 2021), and changes in structural network connectivity between the left putamen nucleus and right caudate nucleus were also correlated with HAMA score (Oosterwijk et al., 2018). It suggested that aberrant ReHo in the contralateral caudate nucleus may disrupt the function of emotional management in PD patients, resulting in anxiety disorders. Researchers also discovered that the ReHo in the right caudate nucleus of PD patients with apathy was lower than that of the normal controls (Sun et al., 2020). And that unlike the excessive worry and tension manifested by anxiety symptoms, apathy usually manifested as indifference and a lack of motivation and emotional response, which further supported that the dysfunction of the caudate nucleus was related to anxiety symptoms in PD patients.

Over the past three decades, clinical neuroimaging investigations have revealed that the cerebellum regulates motor coordination and plays a vital role in the nervous system. Cerebellar dysfunction, particularly posterior cerebellar lobe dysfunction, can cause fear and anxiety in healthy individuals (Lee et al., 2021). In this study, all the pair comparison of the three groups demonstrated reduced ReHo in the right cerebellum. Cerebellum dysfunction in PD patients may contribute to the development of dread and anxiety. Simultaneous engagement of the cerebellum may affect motor and non-motor processes in humans. Other studies discovered that decreased functional connectivity between the putamen nucleus and cerebellum was negatively correlated with HAMA score in PD-A patients (Wang et al., 2017). As a result, the current findings support the idea that abnormal local activities of the brain regions in PD-A patients are interrelated. It also demonstrates that the occurrence of anxiety symptoms in PD patients is due to the dysfunction of coordination between brain regions.

In the current study, compared with HCs, PD-A patients showed increased ReHo in the bilateral precuneus and inferior parietal lobes. The precuneus is associated with self-concentration of attention, self-emotional regulation, and situational memory extraction (Wee et al., 2016), while the inferior parietal lobule is related to the control of visual attention and working memory (Wolters et al., 2019; Xing et al., 2021). A previous study found that increased anxiety was associated with a stronger FC between the amygdala and the precuneus and parietal cortex in PD-A patients (Zhang et al., 2019). Thus, the results of our study indicated that the bilateral precuneus and inferior parietal lobes were also engaged in non-motor processes in PD patients. Nevertheless, the differences were not found in other pairwise comparisons. This may be because the PD patients selected in this study were still in the early stage and the anxiety scores were mostly mild to moderate. Therefore, the ReHo alteration of the bilateral precuneus and inferior parietal lobes between PD-A and PD-NA patients might be inhibited.

There are limitations associated with our study in terms of sample size and research content. Therefore, we will enlarge the sample size and combine ReHo analysis with other analytical methods of rs-fMRI, such as low-frequency amplitude and functional connectivity, to gain a deeper insight into the pathophysiological mechanism underlying PD-A.

## Conclusion

The development of PD-A may be associated with dysfunctional local activities in multiple brain regions, which may serve as neuroimaging markers for the early diagnosis of PD-A. The results of this study suggest that using ReHo analysis to identify functional changes in core regions may advance our understanding of the pathophysiological mechanisms underlying PD-A.

## Data availability statement

The original contributions presented in the study are included in the article/supplementary material, further inquiries can be directed to the corresponding author/s.

## Ethics statement

The studies involving human participants were reviewed and approved by ethics committee of the China-Japan Friendship Hospital. The patients/participants provided their written informed consent to participate in this study. Written informed consent was obtained from the individual(s) for the publication of any potentially identifiable images or data included in this article.

## Author contributions

PZ and KW: conception, design, and administrative support. YZ, YL, and LW: provision of study materials or patients, collection, and assembly of data. YZ and YL: data analysis and interpretation. PZ, KW, YZ, YL, and LW: wrote the manuscript. All authors contributed to the article and approved the submitted version.

## Conflict of interest

The authors declare that the research was conducted in the absence of any commercial or financial relationships that could be construed as a potential conflict of interest.

## Author’s Note

Anxiety is one of the most common non-motor symptoms of Parkinson’s disease (PD)，it is difficult to recognize and diagnose due to modest symptoms and lack of standardized diagnostic criteria. Resting-state functional magnetic resonance imaging (rs-fMRI) has been applied to investigate the neural mechanism of anxiety symptoms in PD, but there was a paucity of studies addressing whether PD-A patients present abnormal local activities. In this prospective study, ReHo analysis was used to explore the alterations of regional activities among PD with anxiety (PD-A) patients, PD without anxiety (PD-NA) patients, and healthy control (HC) subjects. Altered ReHo among the three groups was found in multiple brain regions, including the frontal cortex, cerebella, basal ganglia, and limbic system. Moreover, the increased ReHo values of the right superior frontal gyrus and anterior cingulate gyrus were positively correlated with anxiety scores in PD-A patients. We concluded, the development of PD-A may be associated with dysfunction of these brain regions, which may serve as neuroimaging markers for the early diagnosis of PD-A. In addition, it is suggested that using ReHo analysis to identify functional changes in core regions may advance our understanding of the pathophysiological mechanisms underlying PD-A.

## Publisher’s note

All claims expressed in this article are solely those of the authors and do not necessarily represent those of their affiliated organizations, or those of the publisher, the editors and the reviewers. Any product that may be evaluated in this article, or claim that may be made by its manufacturer, is not guaranteed or endorsed by the publisher.

## Footnotes

^1^ http://rfmri.org/dpabi

^2^ https://www.fil.ion.ucl.ac.uk/spm/software/spm12
